# p62/SQSTM1 Enhances NOD2-Mediated Signaling and Cytokine Production through Stabilizing NOD2 Oligomerization

**DOI:** 10.1371/journal.pone.0057138

**Published:** 2013-02-20

**Authors:** Sangwook Park, Soon-Duck Ha, Macon Coleman, Shahab Meshkibaf, Sung Ouk Kim

**Affiliations:** Department of Microbiology and Immunology and Centre for Human Immunology, Siebens-Drake Research Institute, University of Western Ontario, London, Ontario, Canada; McGill University, Canada

## Abstract

NOD2 is a cytosolic pattern-recognition receptor that senses muramyl dipeptide of peptidoglycan that constitutes the bacterial cell wall, and plays an important role in maintaining immunological homeostasis in the intestine. To date, multiple molecules have shown to be involved in regulating NOD2 signaling cascades. p62 (sequestosome-1; SQSTM1) is a multifaceted scaffolding protein involved in trafficking molecules to autophagy, and regulating signal cascades activated by Toll-like receptors, inflammasomes and several cytokine receptors. Here, we show that p62 positively regulates NOD2-induced NF-κB activation and p38 MAPK, and subsequent production of cytokines IL-1β and TNF-α. p62 associated with the nucleotide binding domain of NOD2 through a bi-directional interaction mediated by either TRAF6-binding or ubiquitin-associated domains. NOD2 formed a large complex with p62 in an electron-dense area of the cytoplasm, which increased its signaling cascade likely through preventing its degradation. This study for the first time demonstrates a novel role of p62 in enhancing NOD2 signaling effects.

## Introduction

NOD2 belongs to a family of 22 different cytosolic pattern recognition receptors known as NOD-like receptors (NLRs) in humans [1]. NOD2 detects muramyl dipeptide (MDP), a unit of peptidoglycan constituting the bacterial cell wall, and induces the production of cytokines and anti-microbial peptides in myeloid cells and Paneth cells, respectively [2], [3]. Mutations in the *NOD2* have shown to be associated with chronic inflammatory diseases such as Blau syndrome and Crohn's disease [4], [5]. The molecular structure of NOD2 contains three distinct domains: two N-terminal caspase-recruitment domains (CARDs), a central nucleotide-binding domain (NBD) and a leucine-rich repeat (LRR) region at the C-terminus. MDP was recently shown to bind to the NBD of NOD2 [6], which likely causes NOD2 homo-dimerization and interaction with the serine/threonine kinase RIP2 (RICK/CARDIAK/RIPK2). RIP2 physically interacts with NOD2 through CARD-CARD homotypic interactions and undergoes K63-linked poly-ubiquitination. Poly-ubiquitination is an integral part of the NOD2 signaling cascade that eventually leads to the activation of the nuclear transcription factor NF-κB through sequentially activating tumor necrosis factor receptor-associated factor (TRAF)6, transforming growth factor-β-activated kinase 1 (TAK1)-binding protein 2, TAK1 and IκB kinase (IKK) [7], [8]. In addition to RIP2, multiple proteins were shown to interact with NOD2 and regulate its downstream signaling events. Some of these proteins include phosphatase 2A [9], ATG16L1 [10], ERBIN (a member of the leucine-rich repeat- and PDZ domain-containing family) [11], [12], guanine nucleotide exchange factor H1 [13], caspase-12 [14], CARD8 [15], A20 [16], TRIM27 [17], TRAF4 [18], GRIM-19 (a protein with homology to the NADPH dehydrogenase complex)[19], IPAF/CLAN/NLRC4 [20], and NALP1 [21]. The multimeric complexes of NOD2 are expected to function as a signaling platform referred to as the “NODosome”, homologous to other NOD-like receptor complexes, such as the “inflammasome” [22] and the “apoptosome” [23].

Autophagy was originally described as an energy homeostasis process that degrades and recycles damaged molecules and organelles through the formation of double-membrane vesicles. Recent studies have further revealed its essential roles in innate immune responses including entrapment/killing of intracellular microorganisms, antigen presentation, and cytokine production [10], [24], [25]. NOD2 has been shown to induce autophagy through a RIP2-dependent manner at least in myeloid and epithelial cells [9], [24], [26]. A portion of NOD2 was also shown to localize at the plasma membrane and recruit the autophagy processing molecule ATG16L1 at the site of bacterial entry, which was a RIP2-independent process [10]. Autophagy can also function as a negative feedback process of inflammatory responses, since it was shown to suppress signaling events induced by Toll-like receptors and NLRP3 [25]. A study also suggested that human peripheral blood mononuclear cells with defects in autophagy resulting from a mutation in *ATG16L1* produced more inflammatory cytokines at the mRNA level when induced by MDP [27], suggesting that autophagy is also involved in NOD2 signaling.

Of the more than 30 different proteins involved in autophagy, p62 (also known as sequestosome-1), is an adaptor protein which sequesters poly-ubiquitinated proteins [28] and *Salmonella*-containing vacuoles [29] to autophagy through interacting with microtubule-associated protein 1 light chain 3 (LC3). In addition to these catabolic roles, p62 has also been shown to regulate various signaling events. For example, it up-regulates signaling events initiated by receptors activated by tumor necrosis factor (TNF)-α, IL-1, nerve growth factor, and RANK-L (receptor activator of NF-κB-ligand) through scaffolding for TRAF6 and atypical protein kinase C with these receptors [30]. In contrast, p62 suppresses Toll-like receptor signaling cascades by inducing MyD88-aggregation and down-regulation of MyD88-TRAF6 complex formation [31]. In light of the multifaceted roles of p62 in autophagy and signal transduction, we examined the role of p62 in NOD2 signaling. This study found that p62 interacted with NOD2 and enhanced its signaling response toward NF-κB activation, and TNF-α and IL-1β production, through stabilizing NOD2 signaling complexes.

## Methods

### Cells and Cell culture

The human monocytic cell line THP-1 (ATCC® TIB-202) was maintained in complete RPMI 1640 medium containing 8% heat-inactivated fetal bovine serum (FBS, Sigma Aldrich), 1 mM MEM non-essential amino acids solution, 1 mM sodium pyruvate, and antibiotics (mixture of 100 U/mL penicillin G sodium, and 100 µg/mL streptomycin sulfate). HEK293T and RAW 264.7 macrophages (kindly provided by Dr. J. Han, The Scripps Research Inst., La Jolla, CA) were cultured in complete DMEM medium containing 8% FBS, and the same reagents as in complete RPMI 1640. Cells were grown at 37 °C in a humidified atmosphere containing 5% CO_2_.

### Plasmids and transfections

pcDNA3-Myc-RIP2 was provided by Dr. Inohara (University of Michigan, Ann Arbor, MI), and plasmids expressing HA-p62 full-length and its mutants (ΔPB1, ΔTRAF6, ΔUBA) were obtained from Dr. Moscat (Sanford/Burnham Institute, La Jolla, CA). Additional deletion mutants (PB1, TRAF6, UBA) and full-length p62 (NM_003900) were subcloned into pEGFP-C1 vector at *Eco*RI and *Bam*H1 sites for co-immunoprecipitation. The full-length human pcDNA3.1-NOD2 cDNA was obtained from Dr. Nunez (University of Michigan, Ann Arbor, MI). NOD2 full-length and mutants (ΔLRR, CARD, LRR, NBD) were further subcloned into pCMV-Myc vector (Clontech) for co-immunoprecipitation, pDsRed-Monomer-C1 or pEGFP (Clontech) for confocal imaging, and the mammalian retroviral expression vector pLNCX (Clontech) for stable transfections into HEK293T cells. TRAF6 cDNA sequences (NM_004620) were cloned and inserted into pCMV-Myc vector for co-immunoprecipitation experiments.

Retrovirus production and cell infection were performed as previously described [32]. Briefly, pLNCX-NOD2 recombinant retroviruses were generated in Phoenix Amphotropic producer cells using the calcium phosphate method of transfection. Viruses were produced at 32°C, and virus-containing medium was collected 24 h post-transfection and filtered through a 0.45 µm filter. HEK293T cells were plated in six-well plates at a density of 5×10^5^ cells/well. One round of retroviral infection was performed by replacing medium with 2 mL of pLNCX-NOD2 virus (containing 4 µg of Polybrene per mL), followed by centrifugation of the six-well plates at 2,000 RPM for 40 min at 32°C. On the third day, culture media were replaced with selection media containing 10 µg/mL of Puromycin (Calbiochem).

### Co-immunoprecipitation for poly-ubiquitination and protein-protein interactions

Twenty-four hours after transfection, cells were washed twice in PBS, overlaid with RIPA lysis buffer (50 mM TrisCl, 150 mM NaCl, 1% Igepal CA-630 (NP-40), 0.5% Sodium Deoxycholate, and 0.1% SDS) and harvested by scraping. Cells were homogenized by pipetting on ice, centrifuged at 12,500 g for 15 min at 4°C, and supernatants were transferred to new 1.5 mL tubes. Ubiquitin-conjugated lysates or total lysates were combined with either anti-Myc or anti-GFP antibodies (Clontech) for 1, 2, 4 h or overnight at 4°C. Twenty microliters of Protein G Sepharose™ Fast Flow Beads (Sigma-Aldrich) were added to lysates and incubated for an additional 1 h. Immunoabsorbents were recovered by centrifugation at 10,000 g for 30 sec and washed five times by resuspension and centrifugation in the same lysis buffer. The immunocomplex was eluted with 4X SDS loading buffer (125 mM Tris-HCl [pH 6.8], 4% SDS, 50% Glycerol, 0.08% Bromophenol Blue, and 5% β-mercaptoethanol).

### Small interfering RNA (siRNA) and small hairpin RNA (shRNA)-mediated silencing

Pre-designed siRNAs directed against human p62 (NM_003900) were purchased from Ambion Life Technologies (ID# s16960; Sense, GGAGCACGGAGGGAAAAGAtt; Antisense,UCUUUUCCCUCCGUGCUCCac). On-TARGETplus SMARTpool mouse p62 (NM_011018) siRNAs were obtained from Thermo Scientific (target sequence; ACAGAUGCCAGAAUCGGAA, CUGCUCAGGAGGAGACGAU, GAACAGAUGGAGUCGGGAA, CCAUGGGUUUCUCGGAUGA). The mouse map1lc3a (mLC3, NM_025735) specific siRNAs were obtained from Ambion, Life technologies. The shRNA-mediated silencing of p62 was performed using calcium phosphate transfection of human p62 specific shRNA constructs (FI336477,-8,-9 and FI336480) and –scramble shRNA (TR30015) (HuSH™, Origene) in THP-1 monocytic cells as described in “Methods” for stable transfection. Transfected THP-1 cells were re-plated with selection media containing 10 µg/mL of puromycin (Calbiochem) to obtain cell colonies stably transfected with p62 shRNA plasmids. For transient gene knock down, HEK293T and RAW 264.7 cells were transfected with scramble siRNA or p62-specific siRNA using Lipofectamine™ RNAiMAX (Invitrogen). Knock down of specific gene products was confirmed by Western blot analysis.

### Confocal microscopy

HEK293T cells were seeded at 0.5×10^6^ cells overnight on coverslips and transfection was performed with GFP-p62, in combination with DsRed-NOD2, DsRed-NBD, DsRed-LRR, or GFP-LC3 and DsRed-NOD2 for 24 h in complete DMEM media at 37°C in a 5% CO_2_ incubator.

The cells were fixed with 4.0% paraformaldehyde in PBS (pH 7.4) at RT for 5 min and rinsed twice with PBS at RT for 5 min. Confocal images were obtained using a Zeiss LSM510 META confocal microscope and analyzed with ZEN software.

### Immunoblot analysis

Total cell lysates were resolved by SDS-PAGE, transferred to PVDF (PALL Life Sciences) or nitrocellulose (Bio-Rad) membranes, and blocked in 5% skim milk in 1 X TBST (0.05% Tween 20). Blots were probed with primary antibodies including anti-human NOD2 [33], anti-GFP (Clontech), anti-Myc (Clontech), anti-HA (Abcam), anti-LC3 (Cell Signaling Technology), anti-p62 (Abnova), anti-phospho p38 (Cell Signaling Technology), anti-NOD2 4A11 [11], [33] and anti-p38 (loading control). Secondary antibodies used included Goat anti-rabbit HRP-conjugate (Thermo Scientific), Goat anti-mouse HRP (Thermo Scientific) or Goat anti-Rat HRP-conjugate antibodies (Jackson ImmunoResearch). Immunoblots were developed using the ECL system (Thermo Scientific).

### NF-κB luciferase assay

HEK293T cells were transfected with pCMV-Myc vector alone, -NOD2 (50 ng of Myc-NOD2), Igk luciferase reporter plasmid (100 ng; kindly provided by Dr. Girardin, University of Toronto, Canada), and β-gal (100 ng). At the same time, gMDP (5 µg/mL, InvivoGen) was added with Lipofectamine™ 2000 (Invitrogen) in the presence or absence of p62-specific siRNA targeting human p62, and then measured 24 h after co-incubation. NF-κB activity was measured with a luciferase reporter detector (Sirius luminometer, Berthold).

### ELISA and TNF-α bioassay

TNF-α levels in cell culture supernatants were measured by ELISA or bioassay. ELISA was followed by manufacturer's instruction (eBioscience). Bioassay for TNF-α concentrations in cell culture supernatants were measured as previously described [34]. Briefly, murine L929 fibroblasts were seeded to each well of a 96-well tissue culture (7×10^4^ cells/well) for 4 h. Cell culture media were then replaced with media containing cyclohexamide (0.3 mg/mL) and the supernatants. Known concentrations of recombinant human TNF-α (eBioscience, 1 mg/mL) were prepared by serial dilution and loaded onto the L929 cells for references. The culture plate was incubated at 37 °C overnight, carefully rinsed with 1X PBS, and then exposed to crystal violet (Sigma-Aldrich) solution for 5 min at RT. Cells were rinsed in PBS twice and crystals were solubilized in 50% acetic acid (Caledon) for 30 min. Optical density was measured using an ELISA plate reader (Bio-Rad) at a wavelength of 570 nm. TNF-α in cell culture supernatants was calculated based on the optical density and known TNF-α references.

### Size-exclusion chromatography

Cells were lysed in RIPA buffer containing 1% Igepal CA-630 (NP-40) and the complete protease inhibitor cocktail (Roche Diagnostics). Lysates were spun at 12,500 RPM for 15 min and supernatants were loaded onto Superdex™ 200 10/300 GL (GE Healthcare, Life Science). Superdex column was equilibrated with 50 mM Tris-HCL (pH 8.0) containing 150 mM NaCl and calibrated with standard proteins (Sigma) containing blue Dextran (2,000 kDa), β-amylase (200 kDa) and bovine serum albumin (66 kDa). Fractions (5 ml) were collected after injecting on to the column and each fraction was analyzed by Western blots for NOD2, p62 and p38.

### Immunogold staining

HEK293T cells were transfected with HA-tagged p62 and GFP-tagged NOD2 using PolyJet™ (SignaGen Laboratories) following manufacturer's instructions. Twenty-four hours post-transfection, cells were harvested in PBS with 1 mM EDTA, and fixed with 3% paraformaldehyde and 0.025% glutaraldehyde in a 0.1 M cacodylate buffer (CAC; pH 7.4) for 2 h. Following fixing, cells were washed in CAC and incubated overnight in the buffer. Cells were enrobed in 5% noble agar the following day, and agar pieces containing cells were dehydrated in 50%, 70%, 85%, 90% and 2×100% ethanol, respectively, for 10 min each. Dehydrated samples were then infiltrated in a 1∶1 mixture of LR White resin: 100% ethanol for 30 min, followed by two infiltrations with pure LR White resin for 90 min and overnight, respectively. Samples were then placed in a gelatine capsule and incubated in an oven at 50°C for 24 h to solidify. The hardened capsule was then cut using an ultramicrotome (Ultracut) into 70 nm thin sections, which were placed onto nickel grids (EM Sciences). Grids were blocked in a 0.2 µm-filtered PBS-BSA buffer (10.4 mM Na_2_HPO_4_, 3.2 mM KH_2_PO_4_, 20 mM NaN_3_, 150 mM NaCl, 1% BSA, pH 7.4) overnight. Blocked grids were then incubated with anti-HA (rabbit; Abcam) and anti-GFP (mouse; Clontech) antibodies at 0.01 mg/mL (1∶100 dilution) and 0.1 mg/mL (1∶10 dilution), respectively, for 2 h at room temperature. Grids were then washed 5 times in PBS-BSA for 5 min each, and probed with anti-rabbit 18 nm colloidal gold (Jackson ImmunoResearch) and anti-mouse 10 nm colloidal gold (Invitrogen) at dilutions of 1∶20 for 1 h. Grids were washed 5 additional times in PBS-BSA for 5 min each, followed by an overnight wash in PBS-BSA. The following day, grids were washed 3 times in 0.2 µm-filtered water before being stained in double 0.2 µm-filtered 2% uranyl acetate for 7 min. After staining, grids were washed 3 times for 1 min each in water and left to dry. Grids were viewed using a Phillips CM10 transmission electron microscope at 60 kV.

## Results and Discussion

### p62 enhances NOD2 signaling in HEK293T cells

**Figure 1:**
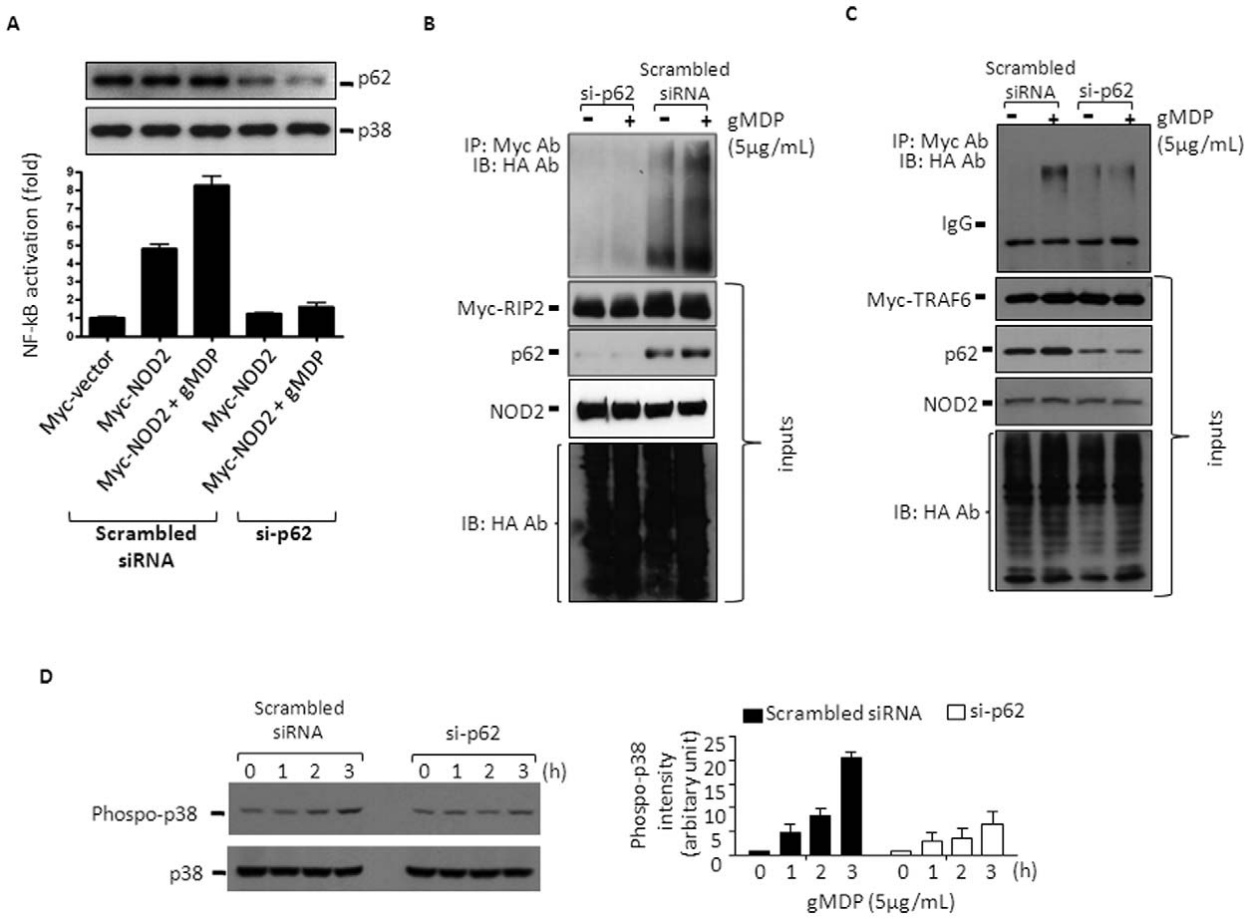
p62 is required for the activation of NF-κB and p38 MAPK, and ubiquitination of RIP2 and TRAF6. **A**. HEK293T cells were transfected with pCMV-Myc-NOD2 and NF-κB luciferase reporter constructs in the presence of scrambled or p62-targeting small interference RNAs (si-p62). Cells were then treated with gMDP (5 µg/mL) for 4 h and NF-κB activity was measured. Data are expressed as the fold of luciferase activity ± SD (n = 3). **B-C**. HEK293T cells stably expressing NOD2 were first treated with scramble siRNA or si-p62 for 24 h, and then transfected with expression vectors for HA-ubiquitin (HA-Ub) and pcDNA3-Myc-RIP2 or pCMV-Myc-TRAF6 for another 24 h. After treating the cells with gMDP (5 µg/mL) for 4 h, RIP2 (B) or TRAF6 (C) was immunoprecipitated with Myc antibodies from total cell lysates and the immune complexes were resolved by SDS-PAGE followed by immunoblotting against HA. Myc-RIP2 or Myc-TRAF6, NOD2 and HA-ubiquitin were analyzed by immunoblot as the inputs (bottom panels). p62 protein levels were also measured by immunoblot. Data shown are representative images of 3 independent experiments. **D**, HEK293T cells stably expressing NOD2 were first treated with scramble siRNA or si-p62 for 24 h, and then treated with gMDP (5 µg/ml) for the times indicated. Activation of p38 was analysed through immunoblotting against tyrosine phosphoryled p38. The ImageJ (NIH) program was used for densitometry analysis of phosphor-p83 bands and data were expressed as mean ± S.D. (n = 3).

We first examined if p62 is involved in NOD2 signaling using an NF-κB-driven luciferase reporter system in HEK293T cells. Cells were treated with p62 or scrambled small interference RNAs (si-p62; Fig. 1A, upper panel), and transiently transfected with Myc-tagged NOD2 and Igκ luciferase reporter plasmids. As shown previously [35], [36], over-expression of NOD2 alone caused about a 5-fold increase in NF-κB reporter activity, which was further increased by the NOD2 ligand N-glycolyl muramyl dipeptide (gMDP) (Fig. 1A, lower panel). However, cells knocked down in p62 failed to respond to NOD2 over-expression alone or gMDP treatments. To further examine the role of p62 in NOD2 signaling, we examined its downstream signaling events: poly-ubiquitination of RIP2 [8], TRAF6 [37] and p38 mitogen-activated protein kinase phosphorylation [38]. For RIP2 and TRAF6 analysis, cells stably transfected with NOD2 were transiently transfected with HA-tagged ubiquitin and Myc-tagged RIP2 (Myc-RIP2) or TRAF6, together with scrambled siRNAs or si-p62. Cells transfected with scrambled siRNAs showed apparent ubiquitinations of RIP2 (Fig. 1B) and TRAF6 (1C) or ubiquitinated proteins co-immunoprecipitated with RIP2 and TRAF6 in response to gMDP; however, cells treated with si-p62 showed no or much less such ubiquitinated proteins. Similarly, treatments of gMDP in cells stably transfected with NOD2 also gradually induced p38 MAPK tyrosine phosphorylation, which was inhibited in cells knocked down in p62 (Fig. 1D). Collectively, these data suggest that p62 enhances NOD2 signaling cascades.

p62 has been shown to recruit signaling molecules for post-translational modifications. For example, p62 recruits the E3 ligase cullin 3 and induces poly-ubiquitination of caspase-8, resulting in protein aggregation and full activation of caspase-8 [39]. Similarly, p62 recruits another E3 ligase, TRAF6, and sustains the activation of NF-κB induced by RANK-ligand [40] and nerve growth factor [41]. NOD2 activation induces K63-linked poly-ubiquitination of RIP2 at K209, resulting in oligomerization of RIP2 and NF-κB activation [8]. To date, the E3 ligase responsible for RIP2 poly-ubiquitination is unknown. Since RIP2 was shown to interact with TRAF1, TRAF5 and TRAF6, but not TRAF2, TRAF3 and TRAF4 [42], it is possible that p62 scaffolds NOD2, RIP2, and TRAF1/5/6 interactions and enhances poly-ubiquitination of RIP2. Therefore, we examined if knocking down p62 affected NOD2 and TRAF6 interaction through performing co-immunoprecipitation experiments. As expected, immunoprecipitation of Myc-TRAF6 also co-precipitated HA-NOD2; however, knocking down p62 had no effects on the level of HA-NOD2 co-precipitation (Supplemental Fig. S1). These results suggest that p62 positively regulates NOD2 signaling, involving events before RIP2 activation.

### NOD2 interacts with p62 through the NBD domain of NOD2 and UBA or TRAF6 domain of p62

**Figure 2:**
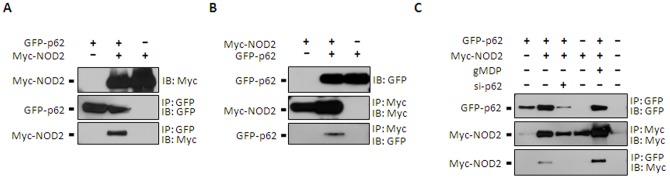
NOD2 physically interacts with p62. **A–C**. HEK293T cells were transiently transfected with expression vectors encoding GFP-tagged p62 (GFP-p62) and/or Myc-tagged NOD2 (Myc-NOD2). After 24 h, total cell lysates were subjected to immunoprecipitation using anti-GFP (A) or anti-Myc (B) antibodies and the immune complexes were resolved by SDS-PAGE followed by immunoblotting against GFP and HA. **C**. Similar experiments as A-B were performed but with or without N-glycorylated muramyldipeptide (gMDP: 5 µg/mL) treatments for 4 h. Data shown are representative images of 3 independent experiments.

To examine if p62 interacts with NOD2, co-immunoprecipitation analyses were performed in HEK293T cells transfected with Myc-NOD2 and GFP-conjugated p62 (GFP-p62) plasmids. As shown in Fig. 2A–B, immunoprecipitations using anti-GFP or anti-Myc monoclonal antibodies were able to co-immunoprecipitate with Myc-NOD2 or GFP-p62, respectively. Also, cells treated with gMDP (5 µg/mL) further enhanced the Myc-NOD2 and GFP-p62 interaction (Fig. 2C), suggesting that p62 interacted with NOD2 better when NOD2 was activated.

**Figure 3:**
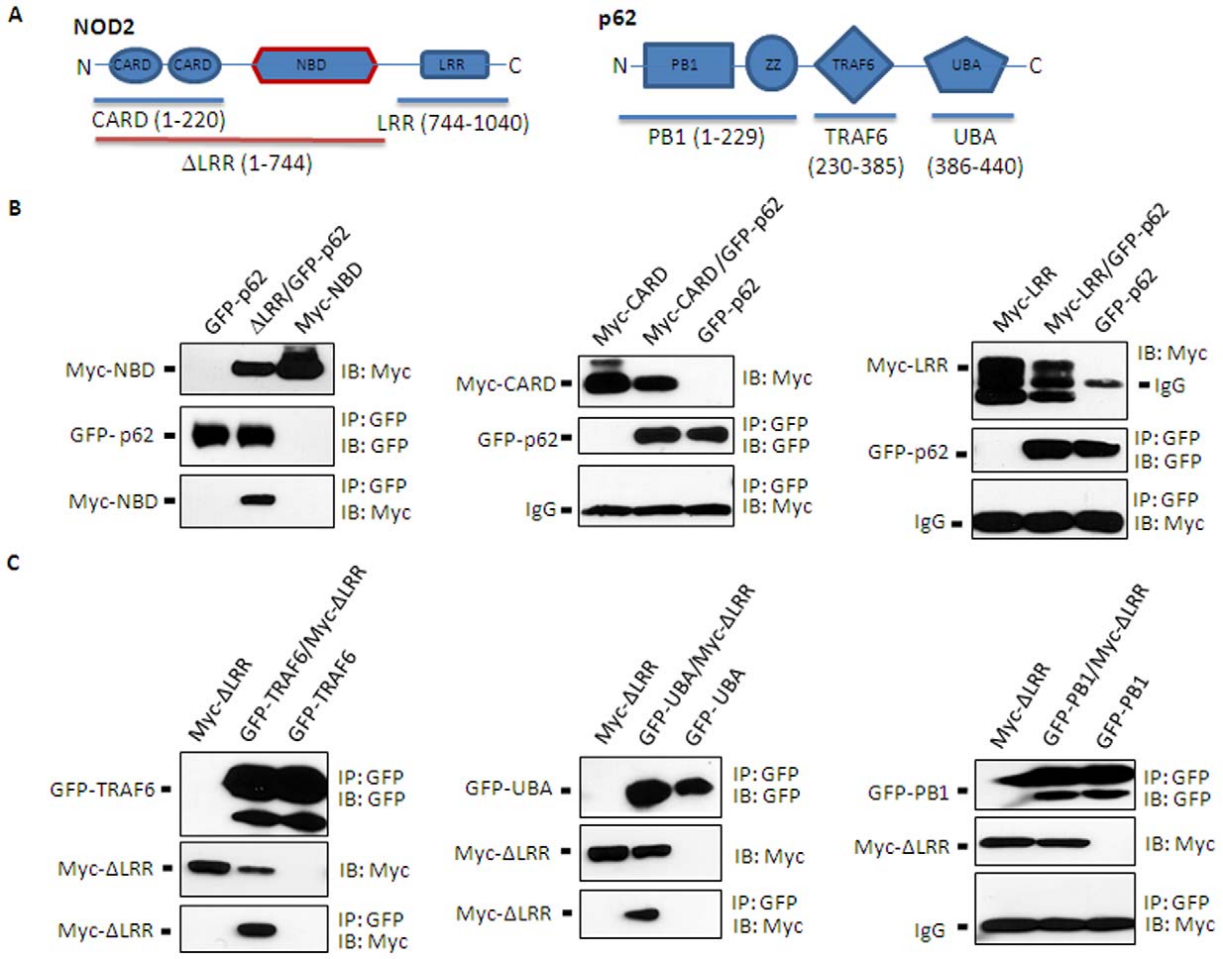
The NBD of NOD2 interacts with both TRAF6 and UBA domains of p62. **A**. NOD2 (left top panel) and p62 (right top panel) structures, and their mutant constructs are schematically presented. **B**. HEK293T cells were transfected with GFP-p62 and Myc-NBD (left middle panel), GFP-p62 and Myc-CARD (center middle panel) or GFP-p62 and Myc-ΔLRR (LRR region-deleted NOD2) (right middle panel). **C**. Similarly, HEK293T cells were transiently transfected with GFP-TRAF6 domain of p62 and Myc-ΔLRR (left bottom panel), GFP-UBA domain of p62 and Myc-ΔLRR (middle bottom panel), and GFP-PB1 domain of p62 and Myc-ΔLRR (right bottom panel); co-immunoprecipitation assays were performed as described in legend to Fig. 2. Data shown are representative images of 3 independent experiments.

To further detail the interaction, different deletion mutants of NOD2 and p62 were examined for their interactions. As aforementioned, NOD2 comprises three distinct motifs: CARD, NBD and LRR (Fig. 3A, left panel). Myc-tagged LRR-deleted NOD2 mutant (Myc-ΔLRR) was strongly co-immunoprecipitated with GFP-tagged p62. However, Myc-tagged proteins containing only CARD (Myc-CARD) or LRR (Myc-LRR) failed to co-immunoprecipitate with GFP-p62 (Fig. 3B). These results suggest that NOD2 interacts with p62 through its NBD domain.

p62 contains at least four distinct motifs (Fig. 3A, right panel): Phox and Bem 1p (PB1), zinc finger (ZZ), TRAF6-binding (TRAF6) and ubiquitin-associated (UBA) domains [43]. The N-terminal PB1 domain is known to accommodate p62 homo-dimerization as well as hetero-dimerization with various signaling molecules including PKCξ/ι/λ, MEKK3, MEK5 and ERK1. ZZ and TRAF6 domains were shown to be involved in the interaction with RIP1 and TRAF6, respectively. The C-terminal UBA domain preferentially binds to K63-linked poly-ubiquitin chains [44] and the LC3-interacting region (LIR), located between UBA and TRAF6 domains, interacts with LC3. Therefore, p62 is expected to function as an autophagy cargo molecule that targets aggregated proteins, cellular organelles and microbes for degradation [45]. We examined how p62 interacted with NOD2 using a similar co-immunoprecipitation approach with different p62 mutants and LRR-deleted NOD2 (ΔLRR) to maximize the interaction. Interestingly, both GFP-TRAF6 and GFP-UBA, but not GFP-PB1, domains were co-immunoprecipitated with Myc-ΔLRR (Fig. 3C). Consistent with these results, ΔLRR was also co-immunoprecipitated with TRAF6 binding domain-deleted (ΔTRAF6) or UBA domain-deleted mutants of p62 (Supplemental Fig. S2). Collectively, these results suggest that the NBD domain of NOD2 interacted with either the TRAF6-binding or UBA domain of p62. We found that NOD2 undergoes both K48- and K63-mediated polyubiquitinations (data not shown), which likely contributes interaction between UBA domain of p62 and NOD2. Further detailed experiments are required to elucidate whether p62 binding to NOD2 through UBA domain requires ubiquitination of NOD2, and how p62 interacting through TRAF6-binding domain and UBA domain affect NOD2 signaling.

### NOD2 is co-localized with p62 in the cytoplasm as a granulated form

**Figure 4:**
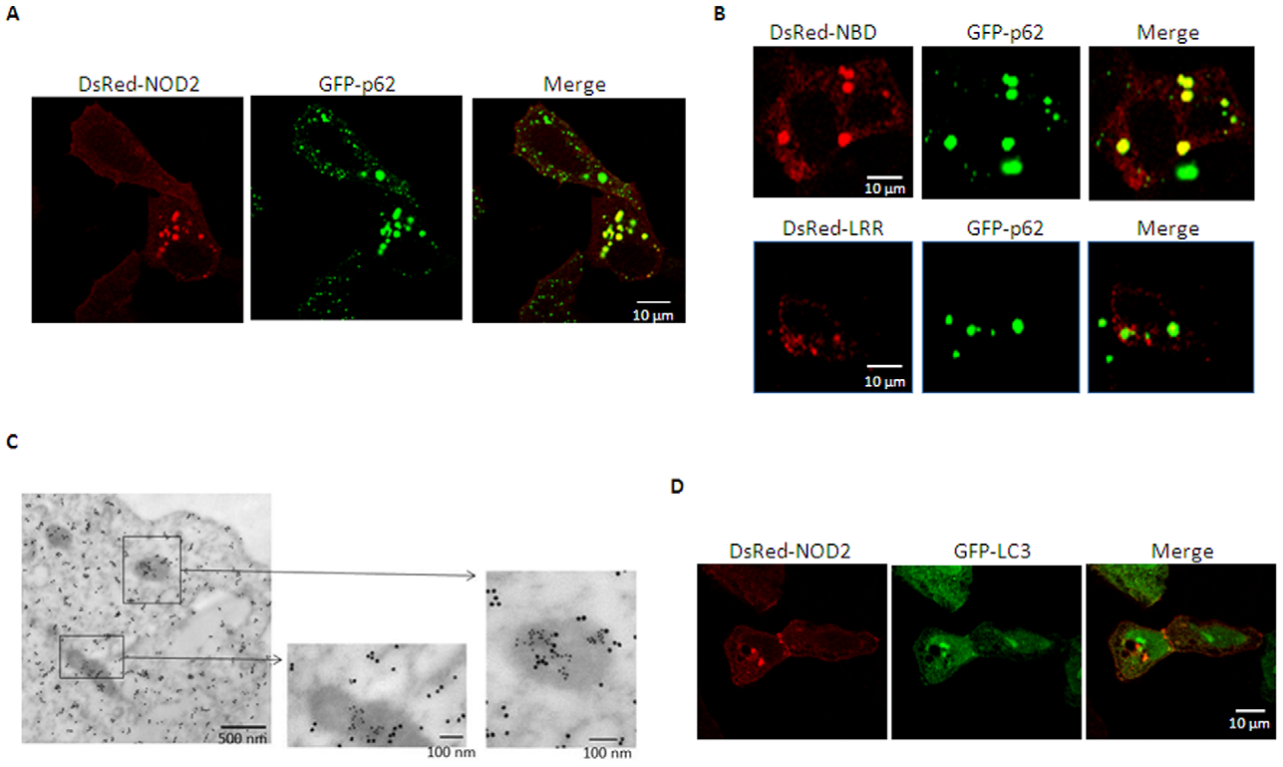
p62 co-localizes with NOD2 through the NBD domain of NOD2. **A**. HEK293T cells were transfected for 24 h with scramble siRNA (left top panel), si-p62 (right top panel), and GFP-NOD2. GFP-NOD2 was visualized using confocal microscopy as described in “Methods”. **B**. Similarly, DsRed-NBD domain, LRR region or full-length NOD2 and GFP-p62 expression vectors were transfected in HEK293T cells and co-localization of these proteins was examined using confocal microscopy. **C**. Immunogold staining of co-localized pCMV-HA-p62 (18 nm colloidal gold) and GFP-NOD2 (10 nm colloidal gold) in HEK293T cells. Cells on grids were viewed using a transmission electron microscope. Scale bars: 500 nm (left bottom), 100 nm (middle, right bottom). **D**. HEK293T cells were transfected with DsRed-NOD2 and GFP-LC3 plasmids. Cells were observed by confocal microscopy and images were acquired using ZEN software.

Previous studies demonstrated that NOD2 could be localized in both the plasma membrane and cytosol as speckles [10], [15], [46]. Indeed, DsRed-NOD2 was localized in both intracellular compartments in punctate form and the plasma membrane in HEK293T cells (Fig. 4A, left panel). Furthermore, cytosolic DsRed-NOD2 positive speckles, but not the plasma membrane associated, were co-localized with p62 (right panel). In line with co-immunoprecipitation results (Fig. 3B), DsRed-conjuated with the NBD of NOD2 also prominently co-localized with GFP-p62 (Fig. 4B, upper lane); whereas, no such co-localization was detected in NOD2 only containing LRR motif (lower lane). To further examine co-localization of these molecules, cells over-expressing both GFP-p62 and HA-tagged full length NOD2 were viewed through EM after immunogold labeling against GFP and HA. As shown in Fig. 4C, aggregated patterns of both GFP-NOD2 (10 nm gold particles) and HA-p62 (18 nm gold particles) were detected in electron-dense areas. However, it could not be determined whether the electron-dense NOD2 and p62-positive areas were autophagosomes, because our immunogold EM staining could not clearly resolve membrane structures. Since p62 associates with autophagosomes through interacting phosphatidylethanolamine conjugated microtubule-associated protein 1 light-chain 3 (LC3) during autophagy [47], [48], [49], we examined whether LC3 co-localized with the cytosolic NOD2-positive granules. LC3-GFP was detected throughout the cytoplasm and as granular forms close to the nucleus. However, DsRed-NOD2-positive granules were localized in distinct locations from those of LC3-GFP (Fig. 4D). These results suggest that cytosolic NOD2-p62 aggregates were not autophagosomes.

Previously, p62 was shown to aggregate with several signaling molecules to enhance their signaling effects. For example, p62 aggregates with poly-ubiquitinated caspase-8 that leads to full activation and processing of the enzyme [39]. p62 is also involved in the formation of large structures known as aggresome-like induced structures (ALIS) [50]. Unlike aggresomes, which are rapidly degraded through a proteasomal route, ALIS are devoid of proteasomes and transient in nature, and recruit ubiquitination enzymes including the ubiquitin-activating enzyme E1, the ubiquitin-conjugating enzyme E2 and the ubiquitin ligase E3 [51], [52]. ALIS were shown to be induced by Toll-like receptors or various stresses [53] and ubiquitinated proteins associated with ALIS were shown to have a much longer half-life than those present in the cytosol [52]. Several features such as the granular aggregation of NOD2 with p62 in non-autophagic vacuoles may point that the electron-dense organelles are ALIS or ALIS-related structures. However, further studies are required to determine if NOD2 is indeed localized in *bona fide* ALIS.

### p62 stabilizes NOD2 oligomerization

**Figure 5:**
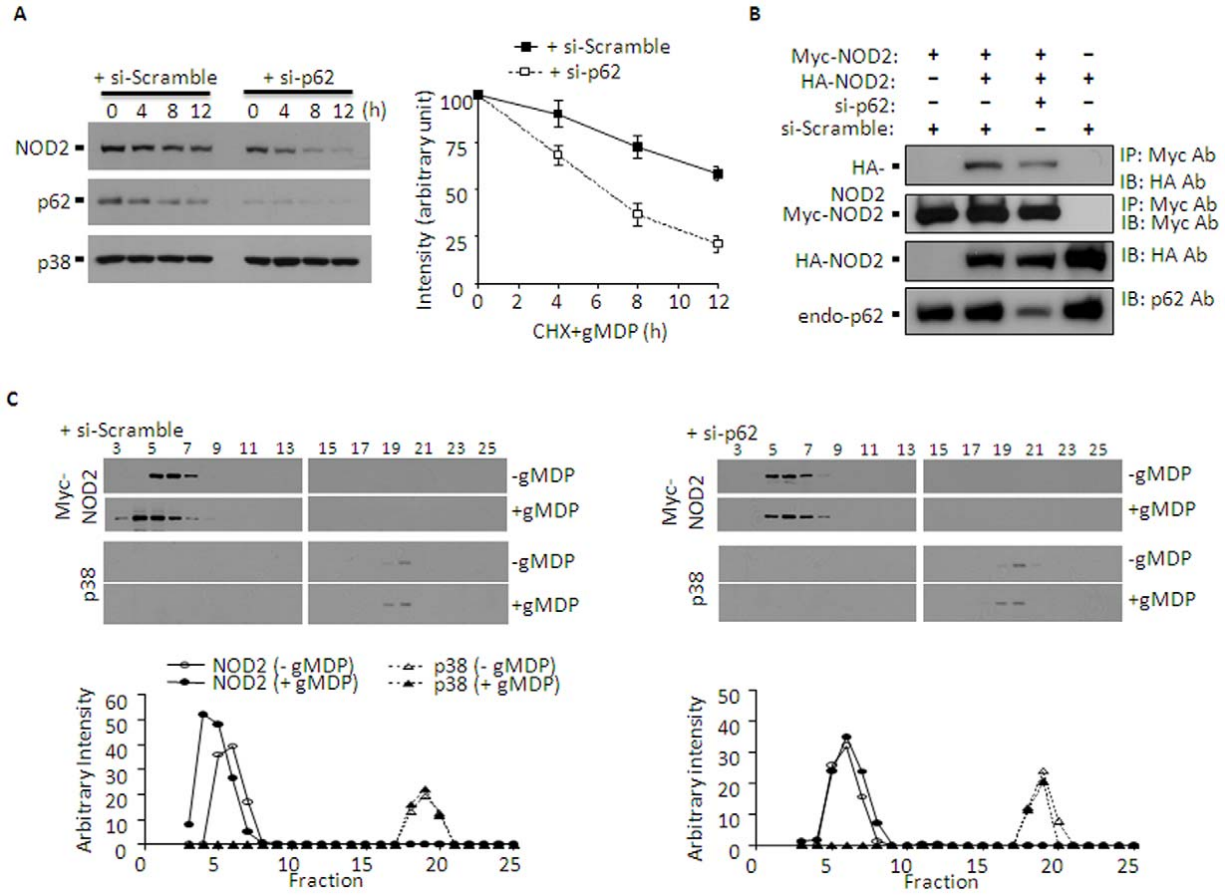
p62 stabilizes gMDP-induced NOD2 oligomers. **A**. HEK293T cells were stably transfected with pLNCX-NOD2 as described in “Methods”. These cells were treated with scramble (si-Scramble) or p62 targeting (si-p62) small interference RNAs for 24 h. Cells were then treated with the translation inhibitor cyclohexamide (CHX, 100 µg/ml) and gMDP (5 µg/ml) for the time indicated, and immunoblots against NOD2 were performed. Intensities of NOD2 bands in comparison with p38 bands (loading control) were expressed as 100% for control samples (right panel). The ImageJ (NIH) program was used for densitometry analysis and data were expressed as mean ± S.D. (n≥4). *p<0.05 (Student t-test). **B**. HEK293T cells were transiently transfected with Myc-NOD2, HA-NOD2, and scramble (si-Scramble) or p62-targeting (si-Scramble) small interference RNAs. Myc-NOD2 was immunoprecipitated with anti-Myc antibodies and immunoblots were performed against HA or Myc. Immunoblots for total lysates against HA and p62 were performed for HA-NOD2 and endogenous p62 inputs (3^rd^ and 4^th^ lanes, respectively). **C**. HEK293T cells were transfected with Myc-NOD2 at 16 h post-transfection with scrambled siRNA or p62-siRNA. After 24 h, cells were further cultured without or with gMDP (5 µg/ml) for 4 h and cell extracts were loaded onto the Superdex™ 200 column. Fractions were analyzed by immunoblot using Myc antibody for estimation of Myc-NOD2 oligomerization (upper panel). Immunoblot for p38 was used as a control. Myc-NOD2 and p38 immunoreactivities were analyzed using NIH ImageJ program (bottom panel).

Considering the multiple roles of p62 in protein modifications (through recruiting E1/E2/E3 proteins or other signaling molecules) and stabilization of proteins through forming ALIS, p62 could have enhanced NOD2 signaling through recruiting TRAF6 to nodosomes or stabilizing NOD2 oligomers. However, knocking down p62 had little effects on recruiting TRAF6 to NOD2 (Supplemental Fig. S1). Thus, we examined whether p62 was involved in stabilization of NOD2 at protein levels. To this end, HEK293T cells stably expressing NOD2 were knocked down in p62 by si-p62 and NOD2 protein levels were examined in the presence of the broad translation inhibitor cyclohexamide (CHX; 100 µg/mL) and gMDP (5 µg/ml). In cells treated with si-Scrambled, both NOD2 and p62, but not p38, gradually degraded over 12 hours (Fig. 5A). However, in cells knocked down in p62, NOD2 degradation was significantly faster. These results are in line with a recent study shown that NOD2 undergoes the 26S proteasome-mediated degradation, which negatively regulates its signaling [17]. Thus, it is possible that p62 leads p62-NOD2 complexes to avoid their degradation by proteasomes. Indeed, the 26S proteasome inhibitor MG132 prevented fast degradation of NOD2 in gMDP-treated p62 knock-down cells (Supplemental Fig. S3). Next, we examined whether p62 also enhanced oligomerization of NOD2. Cells were transiently transfected with both HA- and Myc-conjugated NOD2 expression vectors together with si-Scramble or si-p62, and oligomerization of NOD2 was examined through co-immunoprecipitation. As shown in Fig. 5B, HA-NOD2 was co-immunoprecipitated by anti-Myc antibody, which was diminished by si-p62, suggesting that dimerization or multimerization of NOD2 is formed through a p62-dependent manner. In addition, size-exclusion gel filtration chromatography was used to examine the degree of NOD2 complex formation. Myc-NOD2 was eluted between fraction number 5 and 7 (Fig. 5C, left panels), suggesting that Myc-NOD2 complex was less than 2000 kDa but higher than 200 kDa in size (Supplemental Fig. S4-A). In gMDP-treated cells, Myc-NOD2 was eluted in earlier fractions (number 3-7), suggesting a higher degree of complex formation with about 2000 kDa in size. However, the gMDP-induced Myc-NOD2 complex was not detected in cells treated with si-p62 (Fig. 5C, right panels). Elution of p38 was used as a control and showed no differences in elution patterns between g-MDP or si-p62 treated or non-treated cells. p62 was detected in fractions between 6 and 8, which partially overlapped with those of Myc-NOD2 (Supplemental Fig. S4-B). In the presence of gMDP, p62 was also formed a higher degree of complex which was eluted in fractions 4-9, which fully overlapped with those of Myc-NOD2. Collectively, these results suggest that, in the presence of gMDP, p62 forms a higher degree of complex with NOD2 that may prevent the 26S proteasomal degradation of NOD2.

### p62 is required for cytokine production mediated by NOD2 in macrophages

**Figure 6:**
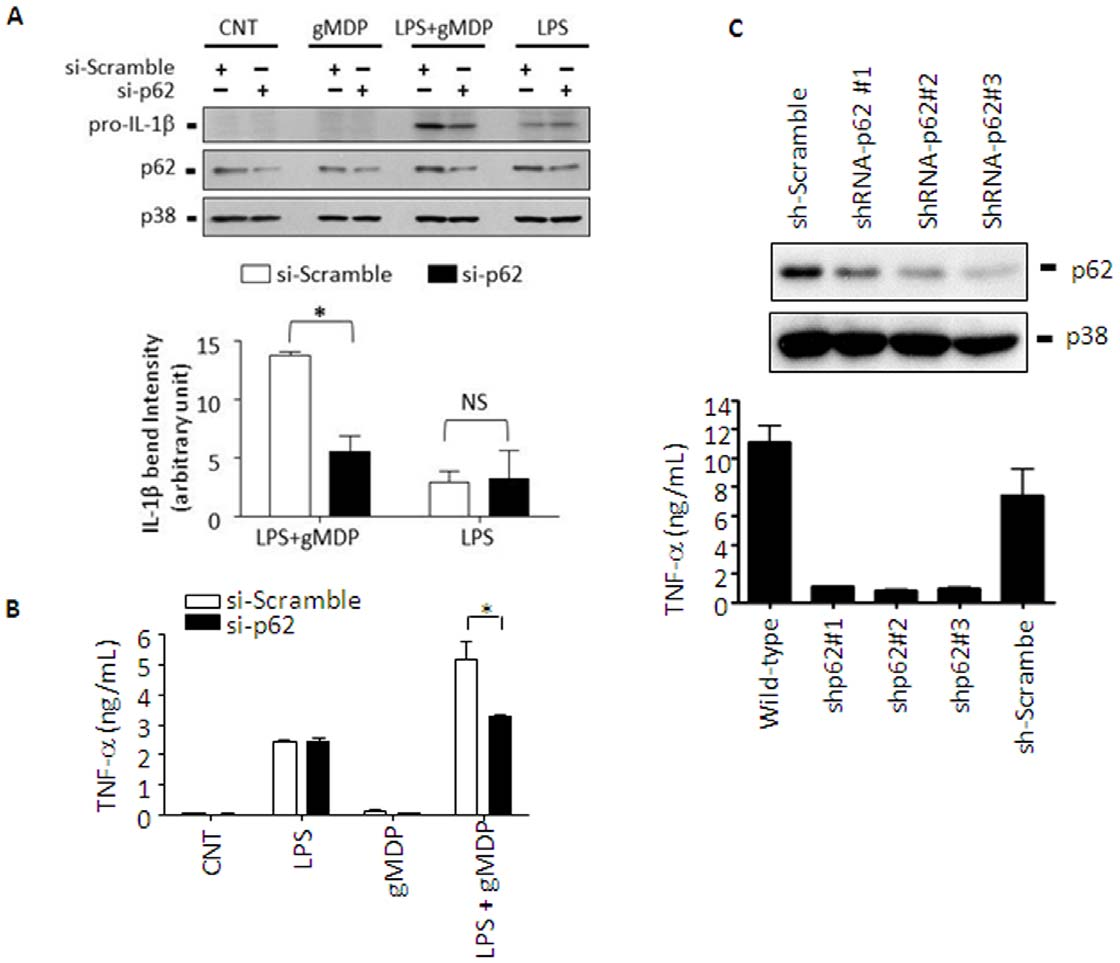
p62 enhances pro-IL-1β expression and TNF-α production in macrophages. **A**. RAW 264.7 cells were transfected with scrambled (si-Scramble) or p62 targeting (si-p62) small interference RNAs using Lipofectamine™ 2000. Twenty four hours post-transfection, cells were treated with a low dose of LPS (50 ng/mL) for 4 h, rinsed with complete media twice, and then incubated with gMDP (5 µg/mL) for another 4 h. Expression of pro-IL-1β was detected using an antibody against IL-1β and p38 was used as a loading control. Densitometric analysis of blots was done using ImageJ (NIH). Data are expressed as mean ± S.D. (n = 3). N.S., not significant; * p<0.05 (Student t-test). **B**. RAW 264.7 cells were transfected with scramble siRNA or p62-siRNA as above A, and cells were treated with LPS (20 ng/mL) for 4 h. After two washes with complete media, cells were further incubated with gMDP (5 µg/mL) for another 4 h and TNF-α concentrations in cell culture media were measured by ELISA according to the manufacturer's instructions (eBioscience). Data are expressed as mean ± S.D. (n = 4); * p<0.05 (Tukey's Multiple Comparison Test). **C**. THP-1 cells were stably transfected with sh-scramble (sh-Scramble) or p62 targeting (sh-p62) small-hairpin RNA producing constructs as described in “Methods”. Three cell clones stably knocked down in p62, pooled sh-Scramble control clones and non-treated wild-type cells were treated with gMDP (5 µg/mL) for 4 h and TNF-α production in the cell culture media was measured using TNF-α bioassay as described in “Methods”.

To confirm the role of p62 in physiologically relevant cell types, we used two macrophage cell lines of murine and human origin: RAW 264.7 (mouse) or THP-1 (human). RAW 264.7 cells express low levels of NOD2 which is rapidly induced by LPS [54]. Consistently, gMDP alone did not induce expression of pro-IL-1β in si-Scramble RNA-transfected RAW 264.7 cells (Fig. 6A). LPS induced pro-IL-1β expression at low levels. In cells pretreated with LPS for 4 h, gMDP significantly enhanced pro-IL-1β expression, as similarly demonstrated before [36], [55]. However, in RAW 264.7 cells transfected with si-p62, no such enhancing effect was detected. Knocking down p62 had no effects on LPS-induced pro-IL-1β expression. In addition, production of TNF-α in response to gMDP was measured in LPS-primed RAW 264.7 cells with or without si-p62. LPS alone induced TNF-α production which was further increased by gMDP (Fig. 6B). However, si-p62 significantly prevented gMDP-induced TNF-α in LPS-primed cells. To further examine the role of p62 in human macrophages, THP-1 cells were knocked down in p62 using small hairpin RNAs (shRNA-p62). Three THP-1 cell clones stably knocked down in p62, pooled clones stably transfected with scrambled sh-RNAs (sh-Scramble), and non-infected wild-type cells were treated with gMDP (Fig. 6B). THP-1 cells responded to gMDP without priming with LPS and induced high levels of TNF-α in wild-type and sh-Scramble transfected clones. However, all three clones knocked down in p62 failed to respond to gMDP. Collectively, these results suggest that p62 was indeed required for optimal IL-1β and TNF production in response to NOD2 in mouse and human macrophages, respectively.

p62 traffics ubiquitinated molecules to autophagosomes through interacting with LC3 [48]. Therefore, we examined the involvement of LC3 in p62-mediated regulation of NOD2. RAW 264.7 cells were knocked down in LC3 using si-RNAs (si-LC3) and pro-IL-1β expression in response to gMDP, LPS and LPS+gMDP were examined. However, knocking down LC3 had no effects on IL-1β production induced by LPS or LPS+gMDP (Supplemental Fig. S5). These results, together with data shown in Fig. 4D suggest that the enhancing effects of p62 in NOD2 stabilization and signaling are not mediated through autophagy.

p62 was first found to be required for NF-κB activation induced by IL-1 [56] or NGF [41], [57] and its sustained activation in RANK (Receptor Activator of Nuclear Factor κ B)-activated osteoclasts [40]. Consistently, p62-deficient mice have defects in sustaining activation of NF-κB in T cells [58]. It was shown that p62 interacts with TRAF6, protein kinase C, MAP kinase kinases and PDK1, which enhances NF-κB and Akt activation [59], [60]. However, p62 was also found to be involved in both positive and negative regulation of NF-κB by interacting with a deubiquitinating enzyme, CYLD [61], [62]. In macrophages, p62 is involved in both TLR- and NLRP3-mediated signaling events. It plays a suppressive role in interferon γ and CpG DNA (TLR9 ligand)-induced cytokine production [63]; whereas, only a partial effect has been detected on TLR4-induced signaling events, namely, activation of the p38 and c-Jun N-terminal kinase but not NF-κB, and production of IL-6 but not TNF [31]. p62 also plays a critical role in NLRP3 inflammasome activation induced by *Mycobacterium abscessus* [64] but at the same time limits NLRP3 inflammasome activation through targeting inflammasomes to autophagy-mediated destruction as a feedback mechanism [65]. Therefore, p62 is involved in multiple signaling cascades with different roles. Here, we demonstrated that p62 plays a positive role in NOD2-mediated signaling cascades probably through forming aggregation of NOD2/p62 oligomers that prevents their degradation. Further studies are required to delineate the mechanism of p62 in preventing degradation of NOD2 and its physiological significance in NOD2 innate immune function.

## Supporting Information

Figure S1 **p62 has no effects on TRAF6 and NOD2 interaction.** HEK293T cells were transiently transfected with pCMV-HA-NOD2 and pCMV-Myc-TRAF6 with or without si-p62 using a PolyJetTM (SignaGen Laboratories). Myc-TRAF6 was immunoprecipitated with anti-Myc antibody (the second lane). HA-NOD2 was co-precipitated with Myc-TRAF6 regardless of the presence of small interference RNAs against p62 (si-p62). Endogenous p62 was knocked down by si-p62 (bottom lane).
(TIF)

Figure S2 **NOD2 interacts with TRAF6 or UBA domain deletion mutants.** **A**. The schematic structure of p62 and mutant constructs are shown. **B**. HEK293T cells were transiently transfected with expression vectors encoding Myc-tagged LRR region deleted NOD2 (Myc-DLRR) and/or different mutants of deletion mutant of p62. Twenty four h post-transfection, total cell lysates were subjected to immunoprecipitation using anti-GFP antibodies and the immune complexes were resolved by SDS-PAGE, followed by immunoblotting against anti-Myc antibodies. Both TRAF6-interacting domain or UBA domain deletion mutants co-immunoprecipitated with NOD2. Data shown are representative images of 3 independent experiments.
(TIF)

Figure S3 **Degradation of NOD2 in p62 knocked down cells was prevented by the 26S proteasome inhibitor MG132.** HEK293T cells stably transfected with pLNCX-NOD2 were treated with si-p62 using a PolyJetTM (SignaGen Laboratories) for 24 h. Cells were then treated with the translation inhibitor cyclohexamide (CHX, 100 µg/ml) and gMDP (5 µg/ml) with or without the 26S proteasome inhibitor MG132 (25 µM) for the time indicated. Stability of NOD2 was analyzed by Western blots using anti-NOD2 (4A11). Western blots for p38 were used as loading controls.
(TIF)

Figure S4 **Size exclusion gel filtration analysis and formation of a higher form p62 complex formaiton by gMDP.** **A**. A mixture of 2000 kDa (Blue dextran; Peak I), 200 kDa (β-amylase; Peak II) and 66 kDa (Bovine serum albumin; Peak III) proteins were eluated through Superdex™ 200 gel filtration column. Elution of standard protiens were detected by UV light. **B**. HEK293T cells were transiently transfected with pCMV-Myc-NOD2 using a PolyJetTM (SignaGen Laboratories) for 24 h. Cells were then treated with gMDP (5 µg/mL) for 4 h and cell extracts were loaded onto the gel filtration column. Elution of p62 was analyzed using Western blots against p62 on each fraction (left panel) and intensities of immuno-reacted bands were ploted (right panel, n = 2). In non-treated cells, p62 complexes were eluted between 2000 kDa-200 kDa fractions; whereas, in gMDP-treated cells, p62 was eluted in ≥ 2000 kDa fractions. These results indicate that gMDP caused a higher degree of p62 complex formation.
(TIF)

Figure S5 **Knocking down LC3 has no effects on p62-mediated NOD2 signaling regulation.** RAW264.7 cells were treated with scrambled- or map1lc3α (LC3)-specific siRNA for 24 hr. Cells were treated with LPS (50 ng/mL) for 4 hr and were rinsed twice with fresh media, followed by a subsequent treatment with gMDP (5 µg/mL) for an additional 4 hr. Total cell lysates were resolved by 14% SDS-PAGE, transferred onto PVDF and blotted with anti-LC3 and anti-IL-1β antibodies.
(TIF)

## References

[1] Proell M, Riedl SJ, Fritz JH, Rojas AM, Schwarzenbacher R (2008) The Nod-like receptor (NLR) family: a tale of similarities and differences. PLoS One 3: e2119. 18446235 10.1371/journal.pone.0002119 PMC2323615

[2] Ting JP, Davis BK (2005) CATERPILLER: a novel gene family important in immunity, cell death, and diseases. Annu Rev Immunol 23: 387–414. 15771576 10.1146/annurev.immunol.23.021704.115616

[3] Girardin SE, Boneca IG, Viala J, Chamaillard M, Labigne A, et al (2003) Nod2 is a general sensor of peptidoglycan through muramyl dipeptide (MDP) detection. J Biol Chem 278: 8869–8872. 12527755 10.1074/jbc.C200651200

[4] Wehkamp J, Harder J, Weichenthal M, Schwab M, Schaffeler E, et al (2004) NOD2 (CARD15) mutations in Crohn's disease are associated with diminished mucosal alpha-defensin expression. Gut 53: 1658–1664. 15479689 10.1136/gut.2003.032805 PMC1774270

[5] Fritz T, Niederreiter L, Adolph T, Blumberg RS, Kaser A (2011) Crohn's disease: NOD2, autophagy and ER stress converge. Gut 60: 1580–1588. 21252204 10.1136/gut.2009.206466 PMC3897479

[6] Mo JY, Boyle JP, Howard CB, Monie TP, Davis BK, et al.. (2012) Pathogen sensing by nucleotide-binding oligomerization domain-containing protein 2 (NOD2) is mediated by direct binding to muramyl dipeptide and ATP. J Biol Chem. 10.1074/jbc.M112.344283 PMC3391102 22549783

[7] Abbott DW, Wilkins A, Asara JM, Cantley LC (2004) The Crohn's disease protein, NOD2, requires RIP2 in order to induce ubiquitinylation of a novel site on NEMO. Curr Biol 14: 2217–2227. 15620648 10.1016/j.cub.2004.12.032

[8] Hasegawa M, Fujimoto Y, Lucas PC, Nakano H, Fukase K, et al (2008) A critical role of RICK/RIP2 polyubiquitination in Nod-induced NF-kappaB activation. EMBO J 27: 373–383. 18079694 10.1038/sj.emboj.7601962 PMC2234345

[9] Homer CR, Kabi A, Marina-Garc IAN, Sreekumar A, Nesvizhskii AI, et al.. (2012) A dual role for receptor interacting protein kinase 2 (RIP2) kinase activity in nucleotide-binding oligomerization domain 2 (NOD2)-dependent autophagy. J Biol Chem. 10.1074/jbc.M111.326835 PMC3408141 22665475

[10] Travassos LH, Carneiro LA, Ramjeet M, Hussey S, Kim YG, et al (2010) Nod1 and Nod2 direct autophagy by recruiting ATG16L1 to the plasma membrane at the site of bacterial entry. Nat Immunol 11: 55–62. 19898471 10.1038/ni.1823

[11] Kufer TA, Kremmer E, Banks DJ, Philpott DJ (2006) Role for erbin in bacterial activation of Nod2. Infect Immun 74: 3115–3124. 16714539 10.1128/IAI.00035-06 PMC1479233

[12] McDonald C, Chen FF, Ollendorff V, Ogura Y, Marchetto S, et al (2005) A role for Erbin in the regulation of Nod2-dependent NF-kappaB signaling. J Biol Chem 280: 40301–40309. 16203728 10.1074/jbc.M508538200

[13] Zhao Y, Alonso C, Ballester I, Song JH, Chang SY, et al (2012) Control of NOD2 and Rip2-dependent innate immune activation by GEF-H1. Inflamm Bowel Dis 18: 603–612. 21887730 10.1002/ibd.21851 PMC3594873

[14] LeBlanc PM, Yeretssian G, Rutherford N, Doiron K, Nadiri A, et al (2008) Caspase-12 modulates NOD signaling and regulates antimicrobial peptide production and mucosal immunity. Cell Host Microbe 3: 146–157. 18329614 10.1016/j.chom.2008.02.004

[15] von Kampen O, Lipinski S, Till A, Martin SJ, Nietfeld W, et al (2010) Caspase recruitment domain-containing protein 8 (CARD8) negatively regulates NOD2-mediated signaling. J Biol Chem 285: 19921–19926. 20385562 10.1074/jbc.M110.127480 PMC2888403

[16] Hitotsumatsu O, Ahmad RC, Tavares R, Wang M, Philpott D, et al (2008) The ubiquitin-editing enzyme A20 restricts nucleotide-binding oligomerization domain containing 2-triggered signals. Immunity 28: 381–390. 18342009 10.1016/j.immuni.2008.02.002 PMC3606373

[17] Zurek B, Schoultz I, Neerincx A, Napolitano LM, Birkner K, et al (2012) TRIM27 negatively regulates NOD2 by ubiquitination and proteasomal degradation. PLoS One 7: e41255. 22829933 10.1371/journal.pone.0041255 PMC3400628

[18] Marinis JM, Homer CR, McDonald C, Abbott DW (2011) A novel motif in the Crohn's disease susceptibility protein, NOD2, allows TRAF4 to down-regulate innate immune responses. J Biol Chem 286: 1938–1950. 21097508 10.1074/jbc.M110.189308 PMC3023490

[19] Barnich N, Hisamatsu T, Aguirre JE, Xavier R, Reinecker HC, et al (2005) GRIM-19 interacts with nucleotide oligomerization domain 2 and serves as downstream effector of anti-bacterial function in intestinal epithelial cells. J Biol Chem 280: 19021–19026. 15753091 10.1074/jbc.M413776200

[20] Damiano JS, Oliveira V, Welsh K, Reed JC (2004) Heterotypic interactions among NACHT domains: implications for regulation of innate immune responses. Biochem J 381: 213–219. 15107016 10.1042/BJ20031506 PMC1133779

[21] Hsu LC, Ali SR, McGillivray S, Tseng PH, Mariathasan S, et al (2008) A NOD2-NALP1 complex mediates caspase-1-dependent IL-1beta secretion in response to Bacillus anthracis infection and muramyl dipeptide. Proc Natl Acad Sci U S A 105: 7803–7808. 18511561 10.1073/pnas.0802726105 PMC2409384

[22] Ogura Y, Sutterwala FS, Flavell RA (2006) The inflammasome: first line of the immune response to cell stress. Cell 126: 659–662. 16923387 10.1016/j.cell.2006.08.002

[23] Zou H, Li Y, Liu X, Wang X (1999) An APAF-1.cytochrome c multimeric complex is a functional apoptosome that activates procaspase-9. J Biol Chem 274: 11549–11556. 10206961 10.1074/jbc.274.17.11549

[24] Cooney R, Baker J, Brain O, Danis B, Pichulik T, et al (2010) NOD2 stimulation induces autophagy in dendritic cells influencing bacterial handling and antigen presentation. Nat Med 16: 90–97. 19966812 10.1038/nm.2069

[25] Harris J (2011) Autophagy and cytokines. Cytokine 56: 140–144. 21889357 10.1016/j.cyto.2011.08.022

[26] Anand PK, Tait SW, Lamkanfi M, Amer AO, Nunez G, et al (2011) TLR2 and RIP2 pathways mediate autophagy of Listeria monocytogenes via extracellular signal-regulated kinase (ERK) activation. J Biol Chem 286: 42981–42991. 22033934 10.1074/jbc.M111.310599 PMC3234870

[27] Plantinga TS, Crisan TO, Oosting M, van de Veerdonk FL, de Jong DJ, et al (2011) Crohn's disease-associated ATG16L1 polymorphism modulates pro-inflammatory cytokine responses selectively upon activation of NOD2. Gut 60: 1229–1235. 21406388 10.1136/gut.2010.228908

[28] Shvets E, Fass E, Scherz-Shouval R, Elazar Z (2008) The N-terminus and Phe52 residue of LC3 recruit p62/SQSTM1 into autophagosomes. J Cell Sci 121: 2685–2695. 18653543 10.1242/jcs.026005

[29] Cemma M, Kim PK, Brumell JH (2010) The ubiquitin-binding adaptor proteins p62/SQSTM1 and NDP52 are recruited independently to bacteria-associated microdomains to target Salmonella to the autophagy pathway. Autophagy 7: 341–345. 10.4161/auto.7.3.14046 PMC3060414 21079414

[30] Salminen A, Kaarniranta K, Haapasalo A, Hiltunen M, Soininen H, et al (2011) Emerging role of p62/sequestosome-1 in the pathogenesis of Alzheimer's disease. Prog Neurobiol 96: 87–95. 22138392 10.1016/j.pneurobio.2011.11.005

[31] Into T, Inomata M, Niida S, Murakami Y, Shibata K (2010) Regulation of MyD88 aggregation and the MyD88-dependent signaling pathway by sequestosome 1 and histone deacetylase 6. J Biol Chem 285: 35759–35769. 20837465 10.1074/jbc.M110.126904 PMC2975200

[32] Miller AD, Buttimore C (1986) Redesign of retrovirus packaging cell lines to avoid recombination leading to helper virus production. Mol Cell Biol 6: 2895–2902. 3785217 10.1128/mcb.6.8.2895-2902.1986 PMC367857

[33] Legrand-Poels S, Kustermans G, Bex F, Kremmer E, Kufer TA, et al (2007) Modulation of Nod2-dependent NF-kappaB signaling by the actin cytoskeleton. J Cell Sci 120: 1299–1310. 17356065 10.1242/jcs.03424

[34] Poltorak A, Smirnova I, He X, Liu MY, Van Huffel C, et al (1998) Genetic and physical mapping of the Lps locus: identification of the toll-4 receptor as a candidate gene in the critical region. Blood Cells Mol Dis 24: 340–355. 10087992 10.1006/bcmd.1998.0201

[35] Inohara N, Ogura Y, Chen FF, Muto A, Nunez G (2001) Human Nod1 confers responsiveness to bacterial lipopolysaccharides. J Biol Chem 276: 2551–2554. 11058605 10.1074/jbc.M009728200

[36] Coulombe F, Divangahi M, Veyrier F, de Leseleuc L, Gleason JL, et al (2009) Increased NOD2-mediated recognition of N-glycolyl muramyl dipeptide. J Exp Med 206: 1709–1716. 19581406 10.1084/jem.20081779 PMC2722178

[37] Abbott DW, Yang Y, Hutti JE, Madhavarapu S, Kelliher MA, et al (2007) Coordinated regulation of Toll-like receptor and NOD2 signaling by K63-linked polyubiquitin chains. Mol Cell Biol 27: 6012–6025. 17562858 10.1128/MCB.00270-07 PMC1952158

[38] Windheim M, Lang C, Peggie M, Plater LA, Cohen P (2007) Molecular mechanisms involved in the regulation of cytokine production by muramyl dipeptide. Biochem J 404: 179–190. 17348859 10.1042/BJ20061704 PMC1868792

[39] Jin Z, Li Y, Pitti R, Lawrence D, Pham VC, et al (2009) Cullin3-based polyubiquitination and p62-dependent aggregation of caspase-8 mediate extrinsic apoptosis signaling. Cell 137: 721–735. 19427028 10.1016/j.cell.2009.03.015

[40] Duran A, Serrano M, Leitges M, Flores JM, Picard S, et al (2004) The atypical PKC-interacting protein p62 is an important mediator of RANK-activated osteoclastogenesis. Dev Cell 6: 303–309. 14960283 10.1016/s1534-5807(03)00403-9

[41] Wooten MW, Geetha T, Seibenhener ML, Babu JR, Diaz-Meco MT, et al (2005) The p62 scaffold regulates nerve growth factor-induced NF-kappaB activation by influencing TRAF6 polyubiquitination. J Biol Chem 280: 35625–35629. 16079148 10.1074/jbc.C500237200

[42] McCarthy JV, Ni J, Dixit VM (1998) RIP2 is a novel NF-kappaB-activating and cell death-inducing kinase. J Biol Chem 273: 16968–16975. 9642260 10.1074/jbc.273.27.16968

[43] Moscat J, Diaz-Meco MT, Wooten MW (2007) Signal integration and diversification through the p62 scaffold protein. Trends Biochem Sci 32: 95–100. 17174552 10.1016/j.tibs.2006.12.002

[44] Seibenhener ML, Babu JR, Geetha T, Wong HC, Krishna NR, et al (2004) Sequestosome 1/p62 is a polyubiquitin chain binding protein involved in ubiquitin proteasome degradation. Mol Cell Biol 24: 8055–8068. 15340068 10.1128/MCB.24.18.8055-8068.2004 PMC515032

[45] Johansen T, Lamark T (2011) Selective autophagy mediated by autophagic adapter proteins. Autophagy 7: 279–296. 21189453 10.4161/auto.7.3.14487 PMC3060413

[46] Barnich N, Aguirre JE, Reinecker HC, Xavier R, Podolsky DK (2005) Membrane recruitment of NOD2 in intestinal epithelial cells is essential for nuclear factor-{kappa}B activation in muramyl dipeptide recognition. J Cell Biol 170: 21–26. 15998797 10.1083/jcb.200502153 PMC2171381

[47] Mizushima N, Levine B, Cuervo AM, Klionsky DJ (2008) Autophagy fights disease through cellular self-digestion. Nature 451: 1069–1075. 18305538 10.1038/nature06639 PMC2670399

[48] Pankiv S, Clausen TH, Lamark T, Brech A, Bruun JA, et al (2007) p62/SQSTM1 binds directly to Atg8/LC3 to facilitate degradation of ubiquitinated protein aggregates by autophagy. J Biol Chem 282: 24131–24145. 17580304 10.1074/jbc.M702824200

[49] Levine B, Kroemer G (2008) Autophagy in the pathogenesis of disease. Cell 132: 27–42. 18191218 10.1016/j.cell.2007.12.018 PMC2696814

[50] Fujita K, Maeda D, Xiao Q, Srinivasula SM (2011) Nrf2-mediated induction of p62 controls Toll-like receptor-4-driven aggresome-like induced structure formation and autophagic degradation. Proc Natl Acad Sci U S A 108: 1427–1432. 21220332 10.1073/pnas.1014156108 PMC3029726

[51] Canadien V, Tan T, Zilber R, Szeto J, Perrin AJ, et al (2005) Cutting edge: microbial products elicit formation of dendritic cell aggresome-like induced structures in macrophages. J Immunol 174: 2471–2475. 15728449 10.4049/jimmunol.174.5.2471

[52] Lelouard H, Ferrand V, Marguet D, Bania J, Camosseto V, et al (2004) Dendritic cell aggresome-like induced structures are dedicated areas for ubiquitination and storage of newly synthesized defective proteins. J Cell Biol 164: 667–675. 14981091 10.1083/jcb.200312073 PMC2172164

[53] Szeto J, Kaniuk NA, Canadien V, Nisman R, Mizushima N, et al (2006) ALIS are stress-induced protein storage compartments for substrates of the proteasome and autophagy. Autophagy 2: 189–199. 16874109 10.4161/auto.2731

[54] Takahashi Y, Isuzugawa K, Murase Y, Imai M, Yamamoto S, et al (2006) Up-regulation of NOD1 and NOD2 through TLR4 and TNF-alpha in LPS-treated murine macrophages. J Vet Med Sci 68: 471–478. 16757890 10.1292/jvms.68.471

[55] Tsai WH, Huang DY, Yu YH, Chen CY, Lin WW (2011) Dual roles of NOD2 in TLR4-mediated signal transduction and -induced inflammatory gene expression in macrophages. Cell Microbiol 13: 717–730. 21199260 10.1111/j.1462-5822.2010.01567.x

[56] Sanz L, Diaz-Meco MT, Nakano H, Moscat J (2000) The atypical PKC-interacting protein p62 channels NF-kappaB activation by the IL-1-TRAF6 pathway. EMBO J 19: 1576–1586. 10747026 10.1093/emboj/19.7.1576 PMC310227

[57] Wooten MW, Seibenhener ML, Mamidipudi V, Diaz-Meco MT, Barker PA, et al (2001) The atypical protein kinase C-interacting protein p62 is a scaffold for NF-kappaB activation by nerve growth factor. J Biol Chem 276: 7709–7712. 11244088 10.1074/jbc.C000869200

[58] Martin P, Diaz-Meco MT, Moscat J (2006) The signaling adapter p62 is an important mediator of T helper 2 cell function and allergic airway inflammation. EMBO J 25: 3524–3533. 16874300 10.1038/sj.emboj.7601250 PMC1538553

[59] Nakamura K, Kimple AJ, Siderovski DP, Johnson GL (2009) PB1 domain interaction of p62/sequestosome 1 and MEKK3 regulates NF-kappaB activation. J Biol Chem 285: 2077–2089. 19903815 10.1074/jbc.M109.065102 PMC2804364

[60] Heo SR, Han AM, Kwon YK, Joung I (2009) p62 protects SH-SY5Y neuroblastoma cells against H2O2-induced injury through the PDK1/Akt pathway. Neurosci Lett 450: 45–50. 19010391 10.1016/j.neulet.2008.11.011

[61] Wooten MW, Geetha T, Babu JR, Seibenhener ML, Peng J, et al (2008) Essential role of sequestosome 1/p62 in regulating accumulation of Lys63-ubiquitinated proteins. J Biol Chem 283: 6783–6789. 18174161 10.1074/jbc.M709496200

[62] Jin W, Chang M, Paul EM, Babu G, Lee AJ, et al (2008) Deubiquitinating enzyme CYLD negatively regulates RANK signaling and osteoclastogenesis in mice. J Clin Invest 118: 1858–1866. 18382763 10.1172/JCI34257 PMC2276399

[63] Kim JY, Ozato K (2009) The sequestosome 1/p62 attenuates cytokine gene expression in activated macrophages by inhibiting IFN regulatory factor 8 and TNF receptor-associated factor 6/NF-kappaB activity. J Immunol 182: 2131–2140. 19201866 10.4049/jimmunol.0802755 PMC4151355

[64] Lee HM, Yuk JM, Kim KH, Jang J, Kang G, et al (2012) Mycobacterium abscessus activates the NLRP3 inflammasome via Dectin-1-Syk and p62/SQSTM1. Immunol Cell Biol 90: 601–610. 21876553 10.1038/icb.2011.72 PMC3389799

[65] Shi CS, Shenderov K, Huang NN, Kabat J, Abu-Asab M, et al (2012) Activation of autophagy by inflammatory signals limits IL-1beta production by targeting ubiquitinated inflammasomes for destruction. Nat Immunol 13: 255–263. 22286270 10.1038/ni.2215 PMC4116819

