# Supplementary figures and images for: p62/SQSTM1 Enhances NOD2-Mediated Signaling and Cytokine Production through Stabilizing NOD2 Oligomerization
Source: PLoS One. 2013 Feb 20;8(2):e57138. doi: 10.1371/journal.pone.0057138 (PMC3577775; doi:10.1371/journal.pone.0057138)

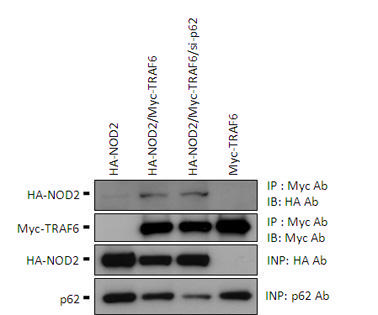

Supplement: Figure S1 — p62 has no effects on TRAF6 and NOD2 interaction. HEK293T cells were transiently transfected with pCMV-HA-NOD2 and pCMV-Myc-TRAF6 with or without si-p62 using a PolyJetTM (SignaGen Laboratories). Myc-TRAF6 was immunoprecipitated with anti-Myc antibody (the second lane). HA-NOD2 was co-precipitated with Myc-TRAF6 regardless of the presence of small interference RNAs against p62 (si-p62). Endogenous p62 was knocked down by si-p62 (bottom lane). (TIF) [file pone.0057138.s001.tif]

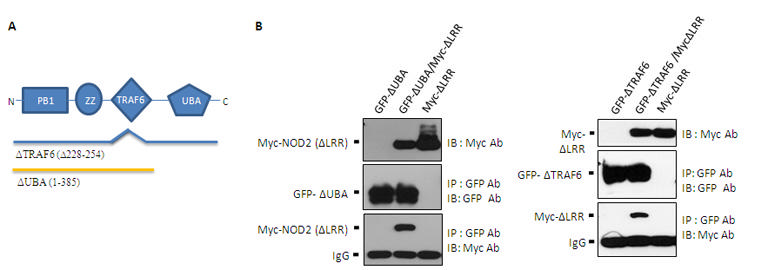

Supplement: Figure S2 — NOD2 interacts with TRAF6 or UBA domain deletion mutants. A. The schematic structure of p62 and mutant constructs are shown. B. HEK293T cells were transiently transfected with expression vectors encoding Myc-tagged LRR region deleted NOD2 (Myc-DLRR) and/or different mutants of deletion mutant of p62. Twenty four h post-transfection, total cell lysates were subjected to immunoprecipitation using anti-GFP antibodies and the immune complexes were resolved by SDS-PAGE, followed by immunoblotting against anti-Myc antibodies. Both TRAF6-interacting domain or UBA domain deletion mutants co-immunoprecipitated with NOD2. Data shown are representative images of 3 independent experiments. (TIF) [file pone.0057138.s002.tif]

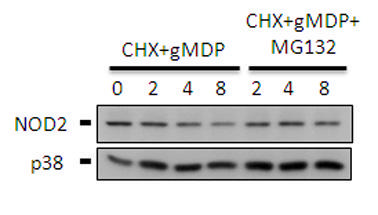

Supplement: Figure S3 — Degradation of NOD2 in p62 knocked down cells was prevented by the 26S proteasome inhibitor MG132. HEK293T cells stably transfected with pLNCX-NOD2 were treated with si-p62 using a PolyJetTM (SignaGen Laboratories) for 24 h. Cells were then treated with the translation inhibitor cyclohexamide (CHX, 100 µg/ml) and gMDP (5 µg/ml) with or without the 26S proteasome inhibitor MG132 (25 µM) for the time indicated. Stability of NOD2 was analyzed by Western blots using anti-NOD2 (4A11). Western blots for p38 were used as loading controls. (TIF) [file pone.0057138.s003.tif]

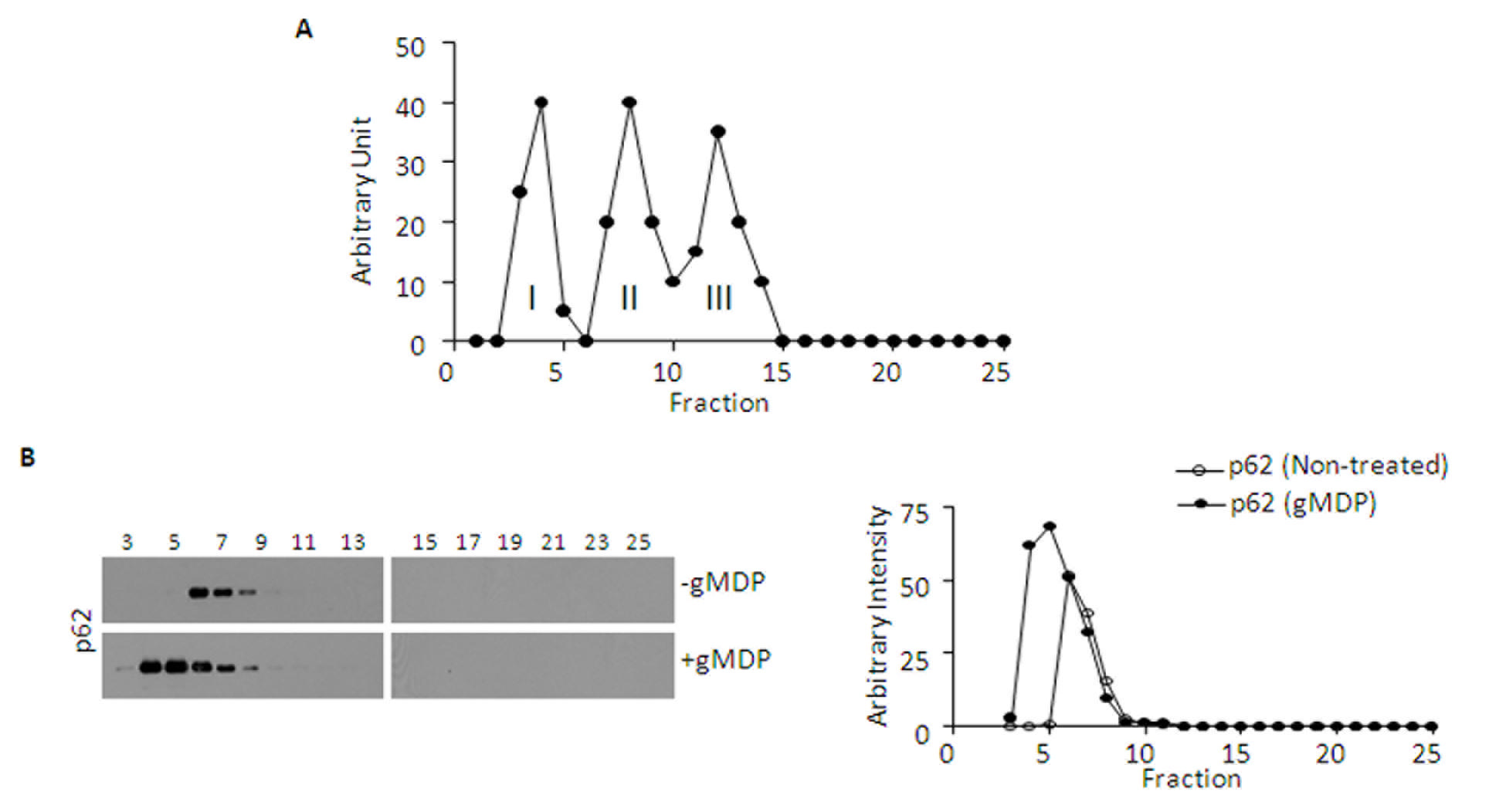

Supplement: Figure S4 — Size exclusion gel filtration analysis and formation of a higher form p62 complex formaiton by gMDP. A. A mixture of 2000 kDa (Blue dextran; Peak I), 200 kDa (β-amylase; Peak II) and 66 kDa (Bovine serum albumin; Peak III) proteins were eluated through Superdex™ 200 gel filtration column. Elution of standard protiens were detected by UV light. B. HEK293T cells were transiently transfected with pCMV-Myc-NOD2 using a PolyJetTM (SignaGen Laboratories) for 24 h. Cells were then treated with gMDP (5 µg/mL) for 4 h and cell extracts were loaded onto the gel filtration column. Elution of p62 was analyzed using Western blots against p62 on each fraction (left panel) and intensities of immuno-reacted bands were ploted (right panel, n = 2). In non-treated cells, p62 complexes were eluted between 2000 kDa-200 kDa fractions; whereas, in gMDP-treated cells, p62 was eluted in ≥ 2000 kDa fractions. These results indicate that gMDP caused a higher degree of p62 complex formation. (TIF) [file pone.0057138.s004.tif]

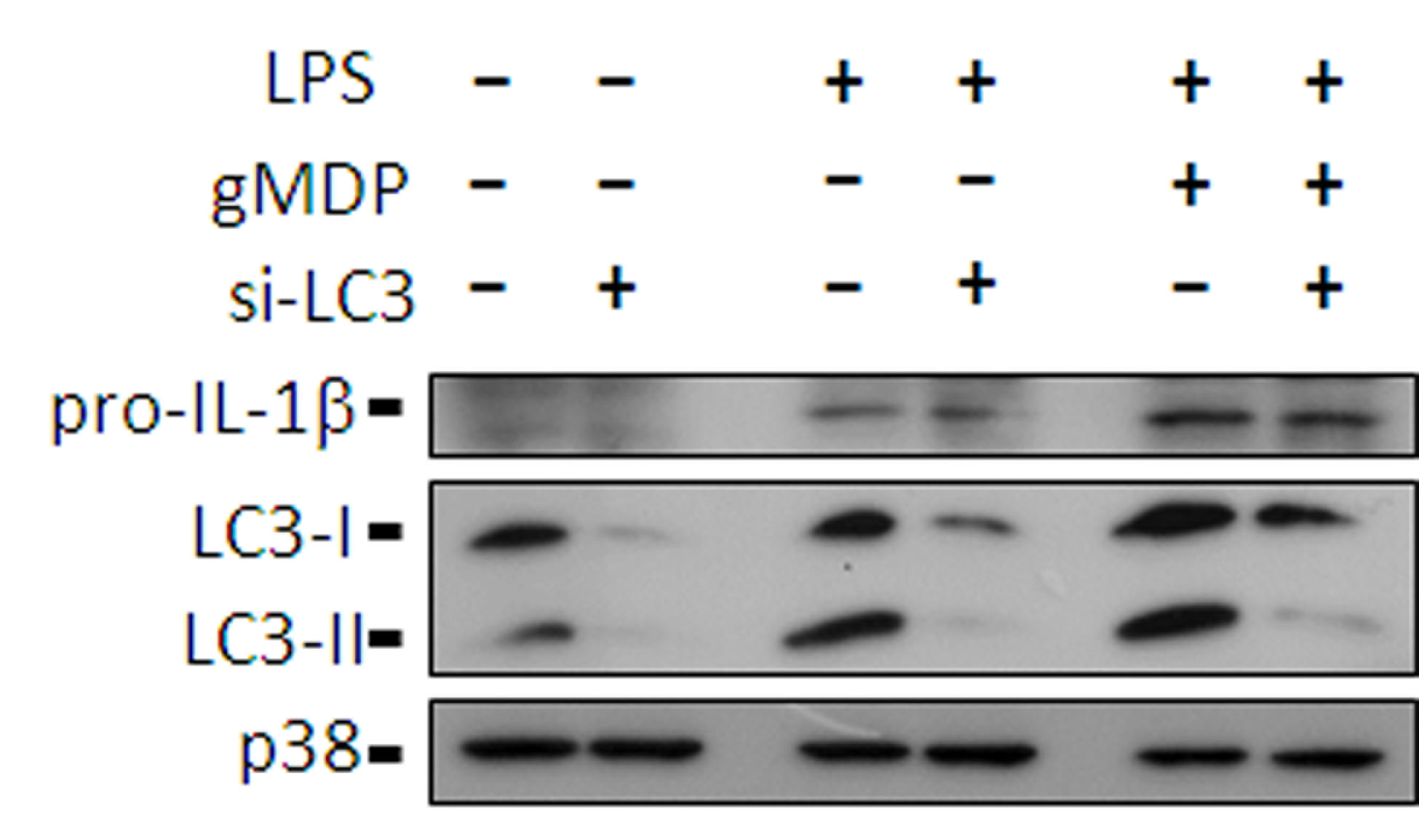

Supplement: Figure S5 — Knocking down LC3 has no effects on p62-mediated NOD2 signaling regulation. RAW264.7 cells were treated with scrambled- or map1lc3α (LC3)-specific siRNA for 24 hr. Cells were treated with LPS (50 ng/mL) for 4 hr and were rinsed twice with fresh media, followed by a subsequent treatment with gMDP (5 µg/mL) for an additional 4 hr. Total cell lysates were resolved by 14% SDS-PAGE, transferred onto PVDF and blotted with anti-LC3 and anti-IL-1β antibodies. (TIF) [file pone.0057138.s005.tif]
